# PNPLA3, TM6SF2, and MBOAT7 Influence on Nutraceutical Therapy Response for Non-alcoholic Fatty Liver Disease: A Randomized Controlled Trial

**DOI:** 10.3389/fmed.2021.734847

**Published:** 2021-10-08

**Authors:** Marcello Dallio, Mario Masarone, Mario Romeo, Concetta Tuccillo, Filomena Morisco, Marcello Persico, Carmelina Loguercio, Alessandro Federico

**Affiliations:** ^1^Department of Precision Medicine, University of Campania “Luigi Vanvitelli”, Naples, Italy; ^2^Department of Medicine and Surgery, University of Salerno, Salerno, Italy; ^3^Department of Clinical Medicine and Surgery, University of Naples Federico II, Naples, Italy

**Keywords:** genetics, insulin resistance, nutraceutics, genome wide association studies, systemic inflammation

## Abstract

**Introduction:** PNPLA3, TM6SF2, and MBOAT7 genes play a crucial role in non-alcoholic fatty liver disease (NAFLD) development and worsening. However, few data are available on their treatment response influence. The aim of this trial is to explore the effect derived from silybin-phospholipids complex (303 mg of silybin-phospholipids complex, 10 μg of vitamin D, and 15 mg of vitamin E twice a day for 6 months) oral administration in NAFLD patients carrying PNPLA3-rs738409, TM6SF2-rs58542926, or MBOAT7-rs641738 genetic variants.

**Materials and Methods:** In all, 92 biopsy-proven NAFLD patients were grouped in 30 NAFLD wild type controls, 30 wild type treated patients, and 32 mutated treated ones. We assessed glycemia (FPG), insulinemia, HOMA-IR, aspartate and alanine aminotransferases (AST, ALT), C-reactive protein (CRP), thiobarbituric acid reactive substance (TBARS), stiffness, controlled attenuation parameter (CAP), dietary daily intake, and physical activity at baseline and end of treatment.

**Results:** The wild-type treated group showed a significant improvement of FPG, insulinemia, HOMA-IR, ALT, CRP, and TBARS (*p* < 0.05), whereas no improvements were recorded in the other two study groups. NAFLD wild type treated patients showed higher possibilities of useful therapeutic outcome (*p* < 0.01), obtained from the prescribed therapeutic regimen, independently from age, sex, comorbidities, medications, CAP, and stiffness in comparison to the mutated group.

**Discussion:** The assessed mutations are independently associated with no response to a silybin-based therapeutic regimen and could be considered as useful predictive markers in this context.

**Clinical Trial Registry Number:** www.ClinicalTrials.gov, identifier: NCT04640324.

## Introduction

Non-alcoholic fatty liver disease (NAFLD) is becoming the most important chronic liver illness and the leading cause of liver transplantation in Western countries (1).

In the last decades, thanks to the growing clinical and epidemiological relevance regarding this topic, the scientific community has focused its attention on the comprehension of the mechanisms sustaining its appearance and worsening to more advanced stages (2–6).

It has been demonstrated that NAFLD onset results from a complex pathogenetic cascade in which a close relationship between environmental and host genetic factors represents the cornerstone for its comprehension that, at the moment, still remains partially unclear (7, 8).

The genome wide association studies (GWAS) have identified the genetic background surrounding NAFLD, counting up to 40 different genetic variants that seem to exert a crucial role in this context until hepatocellular carcinoma (HCC) onset (9, 10). The well-investigated genes involved in this clinical picture are: patatin-like phospholipase domain-containing protein-3 (PNPLA3), the transmembrane 6 superfamily member 2 protein (TM6SF2), and membrane bound O-acyltransferase domain containing 7 (MBOAT7) (11).

PNPLA3 rs738409 C>G (I148C/G-G/G) and the TM6SF2 rs58542926 (167E/K-K/K) C>T genotypes are associated with higher liver fat accumulation and the risk of fibrosis, cirrhosis, and HCC appearance in comparison to the wild-type patients, although the biological reasons surrounding this phenomena are not completely understood (12–14). Similar findings derived from the study of MBOAT7 rs641738 (TMC4C/T-T/T) C>T genetic variant were demonstrated to be linked to the risk of NAFLD worsening in a large population study cohort due to its influence on the hepatic phosphatidylinositol acyl-chain remodeling (15).

Surely, if on one hand the identification of these genetic variants gave to the scientific community important knowledge regarding NAFLD pathogenetic picture, also suggesting a prognostic role in this context, on the other hand, the lack of data concerning their effect on the therapeutic outcome remains an opened question, considering that currently only few a well-designed clinical studies have been published on this topic. The research focused on the most appropriate therapeutic regimen, apart from a hypocaloric well-balanced diet and physical exercise, which currently represents the challenge in this field (16, 17). Over the years, one promising therapeutic regimen has been identified in the use of nutraceuticals, like silybin, the active compound of the silymarin, due to its effect on the improvement of some metabolic parameters that typically are associated with NAFLD evolution (18–20). In a multicenter, phase III, double-blind clinical trial, patients receiving silybin-phospholipids complex showed significant improvement of aspartate and alanine aminotransferase (AST, ALT), gamma-glutamyl-transpeptidase (GGT), and liver histology, represented by steatosis, lobular inflammation, ballooning, and fibrosis improvement, without increase in body weight (21). In this context a deficiency of vitamin D was demonstrated to be related to metabolic disorders pathogenesis like obesity, metabolic syndrome, insulin resistance, and type II diabetes, *via* adipose tissue dysfunction, that, in turn, are recognized as NAFLD-associated comorbidities (22, 23). In the era of the metabolic pandemic associated disorders, the identification of several parameters useful to stratify the prognosis and to predict the therapeutic outcome seems to be essential to design a tailored approach for the correct management of the disease, contributing then to the improvement of the medical assistance in the routine clinical practice.

On the basis of the abovementioned evidence, the aim of this study is to evaluate the effect of PNPLA3 rs738409, TM6SF2 rs58542926, and MBOAT7 rs641738 on 303 mg of silybin-phospholipids complex, 10 μg of vitamin D, and 15 mg of vitamin E twice a day for 6 months administration in NAFLD patients.

## Materials and Methods

### Experimental Design

We performed a baseline comparison of weight, waist-to-height ratio (WHtR), blood pressure measurement, body mass index (BMI), blood glucose and insulin, HOMA-IR, AST, ALT, GGT, blood count, C-reactive protein (CRP), the thiobarbituric acid reactive substance (TBARS), Fibroscan®, and controlled attenuation parameter (CAP) among the three study groups: NAFLD wild type control group (n. 30), NAFLD treated wild type group (n. 30), and NAFLD treated mutated group (n. 32). The block randomization method was used to randomize the 60 not mutated patients in the NAFLD wild type control group and NAFLD treated wild type one, using the online randomization software http://www.graphpad.com/quickcalcs/index.cfm.

The wild-type control group was composed by NAFLD patients without PNPLA3, TM6SF2, and MBOAT3 mutations that did not receive any type of treatment during the study period. The patients inserted in the NAFLD treated wild type and NAFLD treated mutated groups underwent an oral administration of RealSIL 100D® (303 mg of silybin-phospholipid complex, 10 μg of vitamin D, and 15 mg of vitamin E) twice a day for 6 months. In our setting, however, we didn't use vitamin E as a bioactive compound for the obtainment of a kind of therapeutic effect (considering also the very low amount contained in the drug mixture). It is simply inserted in the mixture for a bioengineering reason of molecular stability of the entire drug as a relatively inert substance.

None of the enrolled patients dropped out of the study.

At the end of treatment, the assessment of WHtR, blood pressure measurement, BMI, blood glucose and insulin, HOMA-IR, AST, ALT, GGT, blood count, CRP, TBARS, Fibroscan®, and CAP were reperformed.

During the experimental observation, patients were on a free diet on the basis of dietary habits before the enrollment and any type of physical exercise was recommended during the study period. Food intake was evaluated both at baseline and end of treatment using a software (WinFood, Medimatica s.r.l., Martinsicuro, Italy). We recorded, with a diet diary, the food intake of a complete week, including working days and the weekend. On the basis of the quantities and qualities of food consumed, the program elaborates the daily energy intake and the percentage/caloric number of macronutrients.

For the physical exercise assessment, we submitted a specific questionnaire at baseline and at the end of treatment with some simple questions: Are you doing or have you ever done (in the last 2 years) sport in a continuative and regular way? Have you changed your daily physical activity in the last 6 months? If yes, has it enhanced or worsened?

**Figure 1:**
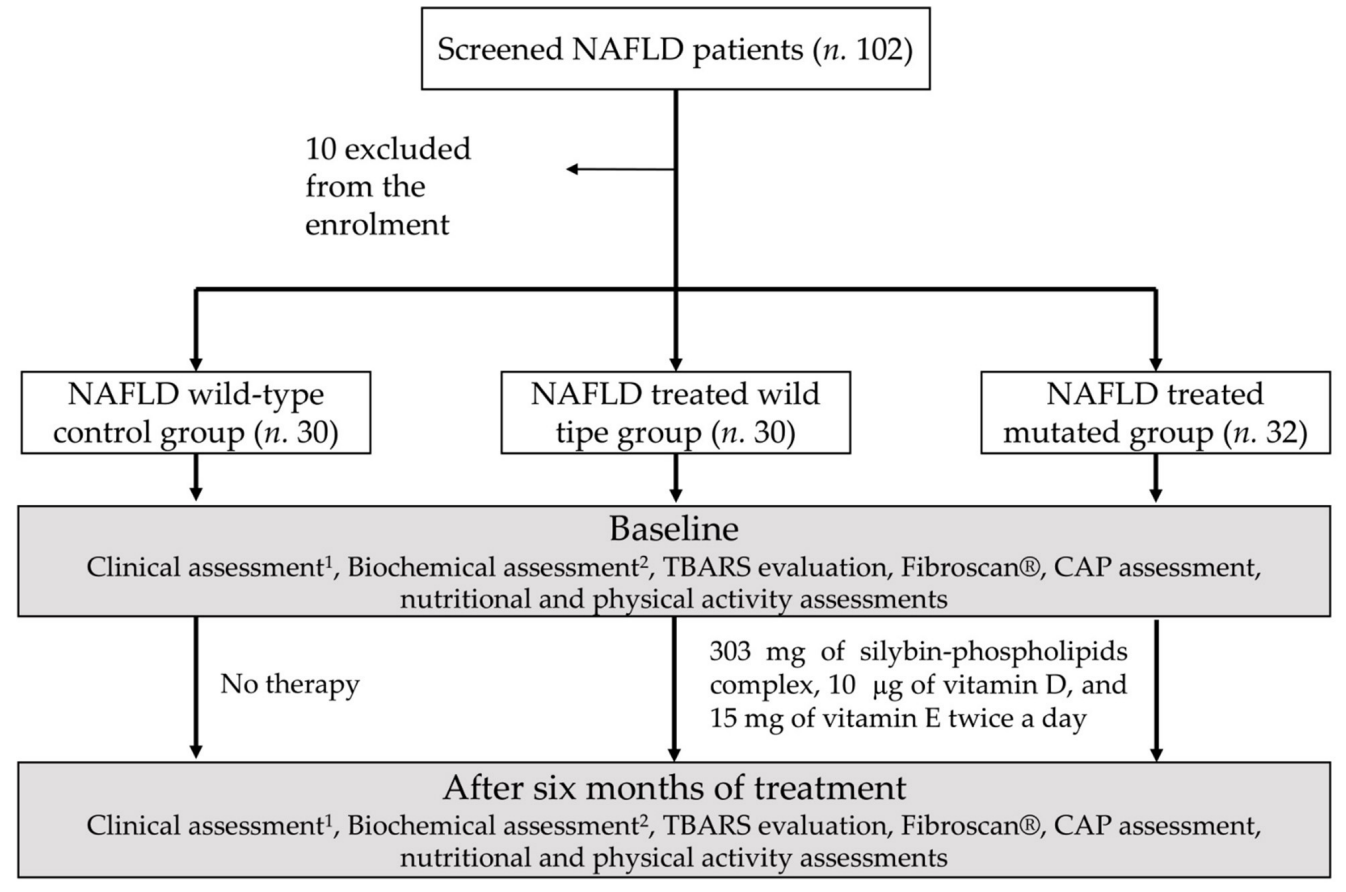
Study design diagram flowchart: schematic representation of the experimental paradigm performed. ^1^Weight, height, and waist-to-height ratio, body mass index, blood pressure measurement, BMI. ^2^Blood glucose and insulin, HOMA-IR, aspartate and alanine aminotransferases, gammaglutamyltranspeptidase, blood count, C-reactive protein.

Alcohol consumption was assessed at the beginning and at the end of the treatment (Figure 1).

The entire study protocol is available on https://www.clinicaltrials.gov.

### Patients

This prospective study is in compliance with ethical guidelines of the Declaration of Helsinki (1975) and has been approved by the ethical committee of the University of Campania “L. Vanvitelli” in Naples. The clinical trial was registered on Clinicaltrials.gov (NCT04640324).

A total of 102 patients with histological diagnosis of NAFLD (obtained during the last 6 months before the enrollment) followed by the Hepato-gastroenterology Division of the University of Campania “Luigi Vanvitelli, between January and October 2017 were screened, after signing an informed consent, for the PNPLA3 rs738409, TM6SF2 rs58542926, and MBOAT7 rs641738 genetic variants and, among them, 32 met the inclusion criteria for the study and showed at least one among PNPLA3 I148I/M, I148M/M, TM6SF2 167E/K, 167K/K, and MBOAT7 TMC4C/T or TMC4T/T genetic variants, and were enrolled together with 60 patients without the mutations. Inclusion criteria were age between 18 and 80 years and diagnosis of NAFLD.

Exclusion criteria were presence of chronic inflammatory disease such as inflammatory bowel disease, rheumatoid arthritis, acute or chronic kidney disease, systemic lupus erythematosus, or other major systemic diseases or tumors, ongoing infections, alcohol or drug abuse history, other etiologies of chronic liver damage, liver cirrhosis or HCC, use of hepatoprotective drugs, and psychological/psychiatric problems that could invalidate the informed consent. So, the patients were divided into three different groups: NAFLD wild type control group, NAFLD treated wild type group, and NAFLD treated mutated group, composed by patients carrying at least one of the abovementioned mutations.

Ten patients were excluded from the enrollment due to the coexistence of several comorbidities and/or advanced stages of liver disease such as cirrhosis and/or hepatocellular carcinoma.

The definition of the presence/absence of NAFLD and the staging were assessed by performing a liver biopsy, serological tests, and collecting clinical data. Medical history, alcohol consumption (AUDIT-C), medications, drug abuse, and smoking habits were also investigated. Blood pressure, weight, and height were directly measured and WHtR was calculated. BMI was also calculated by dividing the weight (kg) by the square of height (m).

After 12 h fast, the patients went to peripheral venous blood sample collection. A complete blood count was done using an automated analyzer (Sysmex, the XP-300™).

Insulin, GGT, and CRP levels were measured enzymatically using commercially available kits (R&D Systems, Minneapolis, MN), AST, ALT, and glucose using colorimetric assay kit (Amplite 13801/13803 and Thermo Fisher Scientific EIAGLUC). HOMA-IR was also calculated using the following formula: fasting insulin (μU/mL) × plasma glucose (mmol/L)/22.5.

### FibroScan and Controlled Attenuation Parameter Assessment

FibroScan® transient elastography (TE) was performed using the FibroScan® version 502 (Echosens, Paris, France) with standard probes (M and XL probes) (24). The XL probe was used when the distance from the skin to the liver capsule, assessed by ultrasonography, exceeded 2.5 cm and/or when BMI was >30. FibroScan® was performed by an expert physician obtaining 10 acceptable measurements (defined as a successful LS measurement), with the maximum number of attempts set at 20. The criteria proposed by Boursier et al. were used to consider the measurement “very reliable” (IQR/M ≤ 0:1), “reliable” (0:1 < IQR/M ≤ 0:3 or IQR/M > 0:3 with LS median <7:1 kPa), or “poorly reliable” (IQR/M > 0:3 with LS median ≥ 7:1 kPa) (24). On the basis of these CAP scores, we classified the enrolled patients in S0, no steatosis (0–10% fat; 0–237 dB/m); S1, mild steatosis (11–33% fat; 238–259 dB/m); S2, moderate steatosis (34–66% fat; 260–292 dB/m); and S3, severe steatosis (>67% fat; ≥293 dB/m) in accordance with the calculation of the attenuation of ultrasonic signals used for TE (25).

### Thiobarbituric Acid Reactive Substance Assessment

TBARS assay was performed using 10 μl of serum. The cromogen TBARS was quantified using a spectrophotometer at a wavelength of 532 nm with 1,1,3,3-tetramethoxyprophane as a standard. The amount of TBARS was expressed as nmol/μg of protein. Presented data are the mean ± standard deviation, resulting from three independent experiments.

### Genomic DNA Extraction From Peripheral Blood Samples

The genomic DNA extraction from peripheral blood samples was performed by using the extraction kit PureLink Genomic DNA Kit (Invitrogen by Life Technologies, U.S.). The DNA amount of each sample was assessed by spectrophotometer (NanoDrop Thermo Fisher Scientific) using a wavelength of 260 nm.

The presence of sample contamination was assessed by using a 280 nm wavelength evaluation and all the sample showed good degrees of purity because the 260/280 ratio was between 1.8 and 2. The DNA extracted was then stored in a −20°C freezer until the analysis of the polymorphisms.

### Real-Time PCR Genotyping

The SNPs analysis, using the access code to the data bank TM6SF2 rs58542926 (SNP 1), MBOAT7 rs641738 (SNP2), PNPLA3 rs738409 (SNP3), was performed using the DNA genotyping RealTime PCR with TaqMan (Applied Biosystem) c_89463510_10 for SNP 1, c_8716820_10 for SNP 2, and c_7241_10 for SNP 3 probes. All the evaluations were done in three phases: DNA PCR amplification, allelic identification, and end point analysis with melting curve.

The first phase was done by using the AmpliTaq Gold® DNA polymerase contained in the TaqMan Universal PCR Master Mix amplifying the target sequence by specific primers contained in the SNP Genotyping Assay 40X together with TaqMan® MGB probes: one marked with fluorochrome VIC® that recognized the sequence of the allele 1 and another probe marked with fluorochrome FAM™ that recognized the sequence of the allele 2. For each experiment, a 48-well plate was used, and three different negative controls were analyzed in order to avoid possible errors due to the contamination of samples. Each experiment was done in triplicate.

The amplifications were performed using the StepOne™ Real Time PCR System (Applied Biosystems).

The genotyping assessment is based on the allelic discrimination thanks to a different fluorescence of the specific gene primer, using two MGB TaqMan® probes, marked at 5' with a different fluorochrome: SNP1 in reverse G in VIC® and A in FAM™; SNIP2 in forward C in VIC® and T in FAM™; and SNIP3 in forward C in VIC® e G in FAM™. For the results analysis the software StepOne™2.0 was used. After the differentiation of the fluorescence made from the probes to the background in each well, it measures the normalized signal intensities (Rn) projecting the results in an allelic discrimination plot.

The software gives to the samples a specific genotype based on the fluorescence signal position: horizontal axis (allele one), vertical axis (allele 2), or diagonal axis (both allele one and two). The prevalence of a specific fluorescence on the other one identified the homozygosis genotype, on the contrary the presence of both the heterozygosis.

### Statistical Analysis

The number of patients (30 in the NAFLD wild type control group, 30 in the NAFLD treated wild type, and 32 in NAFLD treated mutated ones) was calculated using the power and sample size calculation function of STATA-14® for mac-OS, on the basis of an expected difference among the study groups in the response of the HOMA-IR to the therapy, assuming a double amount of patient responders to the therapy in the wild type group in comparison to the mutated one. Specifically, we considered the patients responders if at least one of the following criteria was addressed: normalization of the HOMA-IR (<2.5) at the end of treatment starting from baseline value higher than 2.5; reduction of the HOMA-IR ≥ 2 points at the end of treatment in comparison to baseline.

On the basis of this difference, we estimated 29 patients per arm as the correct sample size of subjects to be investigated maintaining an 0.01 alpha error and a 90% statistical power in a two-sided test with a 95% confidence interval.

A Kolmogorov-Smirnov for normality was performed to evaluate if parametric or non-parametric analysis should be applied. Wilcoxon signed ranks test and *t*-test for dependent groups were performed to compare continuous variables.

The Kruskal-Wallis test or ANOVA test with *post-hoc* Bonferroni analysis, in the case of non-normal or normal distribution, respectively, was performed to compare the continuous variables among the three groups. X^2^s test was performed to compare sex distribution among the three study groups.

Pearson's or Kendall Tau-b correlations as well as linear regression were applied to test the associations among variables. Multiple logistic regression analysis was performed to assess the relationship between the genotype of patients (NAFLD treated wild type vs. mutated patients) and the therapeutic outcome on insulin, HOMA-IR, ALT, CRP, and TBARS.

We identified the following values as the specific cut-offs to consider the parameter improved: insulin normalization (<24 micro-IU/ml), normalization of the HOMA-IR (<2.5) at the end of treatment starting from baseline values higher than 2.5 and/or reduction of the HOMA-IR ≥ 2 points at the end of treatment in comparison to baseline, ALT normalization (<45 IU/L), CRP normalization (<0.6 mg/dL) or reduction of at least 1 mg/dL, and TBARS reduction of at least 10 nmol/μg. The abovementioned parameters were chosen in relation to the main therapeutic effect of silybin in this context.

The relative risk (RR) of a useful therapeutic response was calculated considering the confounding variables (age, sex, comorbidities, medications, liver stiffness, and CAP) and in each of these separated logistic regressions, we adjusted for the baseline measurement of the outcome evaluation. Statistical significance was defined as *p* < 0.05 in a two-tailed test with a 95% confidence interval.

All continuous variables were expressed as mean ± standard deviation/standard error. Statistical analyses were performed using Statistical Program for Social Sciences (SPSS®) vs.18.0.

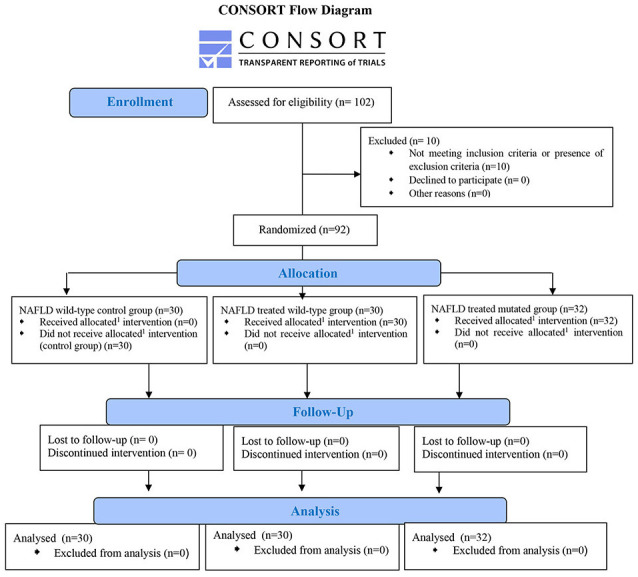



^1^Allocated intervention: 303 mg of silybin-phospholipids complex, 10 μg of vitamin D, and 15 mg of vitamin E twice a day.

## Results

### Dietary Habits, Physical Exercise, and Baseline Comparison Among Study Groups

None of the enrolled patients showed an alteration of the AUDIT C assessment both at baseline and at end of treatment. Moreover, none of them have regularly and continuously done physical exercise in the last 2 years before the enrollment as well as modified the physical activity during the study period. No significant differences were highlighted among the three study groups regarding the daily nutritional intake at baseline and end of treatment (Supplementary Table 1).

Six patients affected by diabetes mellitus were enrolled in the NAFLD wild type control group, 9 in the NAFLD treated wild type, and 7 in the NAFLD treated mutated ones. None of them assumed insulin for the diabetes treatment. Only two of them in the NAFLD wild type control group, and three in both NAFLD treated groups assumed metformin. Among the study groups we enrolled 35 patients affected by arterial hypertension treated with ACE-inhibitors, calcium antagonists, or non-selective beta blocker: 13 in the NAFLD wild type control group, 8 in the NAFLD treated wild type, and 14 in the NAFLD treated mutated one, respectively. Two of the enrolled patients, both of them in the NAFLD treated wild type group, were affected by paroxysmal atrial fibrillation treated with warfarin.

The baseline characteristics of the study population are summarized in Table 1.

**Table 1:** Effect of the therapy on clinical, biochemical, thiobarbituric acid reactive substance, Fibroscan® and controlled attenuation parameter assessment among the three study groups.

**Variables (M ± SD)**	**NAFLD wild type control group (n. 30)**			**NAFLD wild type treated group (n. 30)**			**NAFLD mutated treated group (n. 32)**			***p*-value of the baseline comparison among the groups**
	**Baseline**	**End of treatment**	***p***	**Baseline**	**End of treatment**	***p***	**Baseline**	**End of treatment**	***p***	
Age (*y*)	47.9 ± 14.2	**/**	**/**	45.2 ± 15	**/**	**/**	47.1 ± 13	**/**	**/**	Mutated vs. WT treated: >0.999 Mutated vs. WT control: >0.999 WT treated vs. WT control: >0.999
Sex (M/F)	15/15	**/**	**/**	13/17	**/**	**/**	18/14	**/**	**/**	0.597
BMI (kg/m^2^)	28.1 ± 2.7	28.1 ± 2.7	0.827	29.09 ± 3.1	29.06.8 ± 3.1	0.871	30.5 ± 4.1	31.6 ± 3.6	0.062	Mutated vs. WT treated: >0.999 Mutated vs. WT control: 0.108 WT treated vs. WT control: 0.694
WHtR	0.9 ± 0.13	0.91 ± 0.12	0.112	0.96 ± 0.13	0.95 ± 0.12	0.679	1.08 ± 0.25	1.08 ± 0.26	0.372	Mutated vs. WT treated: 0.488 Mutated vs. WT control: **0.005** WT treated vs. WT control: 0.274
Metavir	1.4 ± 0.56	**/**	**/**	1.46 ± 0.57	**/**	**/**	1.46 ± 0.67	**/**	**/**	Mutated vs. WT treated: >0.999 Mutated vs. WT control: >0.999 WT treated vs. WT control: >0.999
NAFLD activity score	5.36 ± 1.21	**/**	**/**	5.46 ± 1.27	**/**	**/**	5.93 ± 1.16	**/**	**/**	Mutated vs. WT treated: 0.51 Mutated vs. WT control: 0.2 WT treated vs. WT control: >0.999
CAP (dB/m)	271.9 ± 35.8	272.8 ± 36.8	0.373	271.5 ± 51.4	263.9 ± 43.6	0.251	316.7 ± 35.7	305.4 ± 43.4	0.136	Mutated vs. WT treated: **0.0002** Mutated vs. WT control: **0.0002** WT treated vs. WT control: >0.999
Stiffness (kPa)	4.7 ± 1.5	4.9 ± 1.2	0.117	4.3 ± 0.7	4.4 ± 0.7	0.568	4.8 ± 1.5	4.7 ± 1.7	0.893	Mutated vs. WT treated: 0.711 Mutated vs. WT control: >0.999 WT treated vs. WT control: >0.999
SBP (mmHg)	126 ± 14	126 ± 11	0.664	126 ± 13	127 ± 11	0.436	129 ± 15	130 ± 11	0.299	Mutated vs. WT treated: 0.981 Mutated vs. WT control: >0.999 WT treated vs. WT control: >0.999
DBP (mmHg)	74 ± 7	73 ± 5	0.243	75 ± 8	75 ± 7	0.279	76 ± 9	77 ± 8	0.492	Mutated vs. WT treated: >0.999 Mutated vs. WT control: 0.811 WT treated vs. WT control: >0.999
FPG (mg/dl)	99.7 ± 11.7	101 ± 14	0.503	100.3 ± 24.5	89.7 ± 18.5	**0.0009**	110.9 ± 24	110.9 ± 26.3	0.721	Mutated vs. WT treated: 0.464 Mutated vs. WT control: 0.455 WT treated vs. WT control: >0.999
Insulinemia (μU/ml)	25 ± 9	25.2 ± 9	0.464	25.7 ± 8.8	19.4 ± 9	**0.006**	26.4 ± 6.2	25.2 ± 6.8	0.447	Mutated vs. WT treated: >0.999 Mutated vs. WT control: 0.505 WT treated vs. WT control: >0.999
HOMA-IR	6.15 ± 2.38	6.33 ± 2.71	0.545	6.59 ± 3.26	4.51 ± 3.13	**0.0001**	7.35 ± 2.91	7.16 ± 3.46	0.742	Mutated vs. WT treated: >0.999 Mutated vs. WT control: 0.258 WT treated vs. WT control: >0.94
GGT (IU/L)	52 ± 50	58 ± 61	0.161	59 ± 31	57 ± 30	0.281	63 ± 77	64 ± 68	0.657	Mutated vs. WT treated: >0.999 Mutated vs. WT control: >0.999 WT treated vs. WT control: >0.999
AST (IU/L)	31 ± 13	29 ± 16	0.322	34 ± 20	34 ± 14	0.45	39 ± 34	34 ± 31	0.571	Mutated vs. WT treated: >0.999 Mutated vs. WT control: >0.999 WT treated vs. WT control: >0.999
ALT (IU/L)	54 ± 28	56 ± 37	0.81	57 ± 28	34 ± 14	**<0.0001**	68 ± 32	68 ± 43	0.674	Mutated vs. WT treated: 0.68 Mutated vs. WT control: 0.232 WT treated vs. WT control: >0.999
CRP (mg/dl)	3.26 ± 2.04	3.46 ± 2.36	0.703	3.72 ± 2.67	2.1 ± 2.37	**0.0005**	4.38 ± 2.28	4.04 ± 2.51	0.814	Mutated vs. WT treated: 0.144 Mutated vs. WT control: **0.041** WT treated vs. WT control: >0.999
TBARS (nmol/μg)	21.12 ± 23.35	23.81 ± 23.01	**0.001**	22.75 ± 25.91	10.31 ± 8.83	**0.002**	24.62 ± 15.15	23.25 ± 18.39	0.462	Mutated vs. WT treated: 0.2 Mutated vs. WT control: 0.1 WT treated vs. WT control: >0.999

*BMI, body mass index; WHtR, waist-to-height ratio; CAP, controlled attenuation parameter; SBP, systolic blood pressure; DBP, diastolic blood pressure; FPG, fasting plasma glucose; HOMA-IR, homeostatic model assessment for insulin resistance; GGT, gamma-glutamyl transferase; AST, aspartate aminotransferase; ALT, alanine aminotransferase; CRP, C reactive protein; TBARS, thiobarbituric acid reactivesubstance*.

*For the comparison of the therapeutic outcome in each group for the continuous variables, Wilcoxon signed ranks test and t-test for dependent groups were performed according to non-normal and normal distribution, respectively. The Kruskal-Wallis test or ANOVA test with post-hoc Bonferroni analysis, in the case of non-normal or normal distribution, respectively, were performed to compare the continuous variables among three groups. X^2^-test was performed to compare sex distribution among the three study groups. The bold was used to indicate significative p values <0.05*.

As noticed, we found a statistically significant difference among the study groups regarding WHtR, higher in NAFLD treated mutated group in comparison to NAFLD wild type controls (*p* = 0.005) CAP, higher in NAFLD treated mutated group in comparison to NAFLD wild type controls and NAFLD wild type treated group (*p* = 0.0002 for both), CRP, higher in NAFLD treated mutated group in comparison to NAFLD wild type controls (*p* = 0.045).

### Therapeutic Outcome

By analyzing the therapeutic outcome obtained from 6 months oral administration of 303 mg of silybin-phospholipid complex, 10 μg of vitamin D, and 15 mg of vitamin E twice a day we found some differences among the groups. The wild type one demonstrated a significant improvement, in comparison to the baseline, of several evaluated parameters assessed at the end of the treatment: FPG, insulinemia, HOMA-IR, ALT, CRP, TBARS (*p* = 0.0009, 0.006, 0.0001, <0.0001, 0.0005, 0.002, respectively), differently the NAFLD wild type control group showed a statistically significant worsening of TBARS levels at the end of treatment in comparison to baseline (*p* = 0.001), whereas the mutated one did not show any statistically significant variation of all the clinical, biochemical, and stiffness/CAP assessments (Table 1).

The multiple logistic regression models showed that NAFLD wild type treated patients had higher possibilities of useful therapeutic outcome on insulinemia (RR: 6.876, 95% CI: 1.748–27.042, *p* = 0.006), HOMA-IR (RR: 11.341, 95% CI: 2.687–47.857, *p* = 0.001), ALT (RR: 7.198, 95% CI: 1.755–29.530, *p* = 0.006), CRP (RR: 12.254, 95% CI: 2.627–57.166, *p* = 0.001), and TBARS (RR: 6.912, 95% CI: 1.693–28.210, *p* = 0.007) in comparison to the patients carrying at least one of the abovementioned mutations.

### Per Number of Mutation Sub Analysis

As additional analysis, we further differentiated in the mutated group: patients with one single mutation (n. 13), patients with two (n. 13), and three (n. 6) mutations, independently of the homozygosis or heterozygosis for the assessed allelic variants. Specifically, we enrolled in a single mutation group 8 patients with PNPLA3 allelic variant (n. 4: I148I/M and 4 I148M/M), 3 TM6SF2 mutated patients (n. 1: 167E/K and 2: 167K/K), 2 MBOAT7 mutated patients (n. 1 TMC4 C/T and 1 TMC4 T/T); in two mutations group we enrolled 4 patients with PNPLA3 and MBOAT7 allelic variants (n. 3: I148I/M and 1 I148M/M, n. 2 TMC4 C/T and 2 TMC4 T/T), 6 PNPLA3 and TM6SF2 patients (n. 3: I148I/M and 3 I148M/M, n. 4: 167E/K and 2: 167K/K), 3 TM6SF2 and MBOAT7 mutated patients (n. 1: 167E/K and 2: 167K/K, n. 1 TMC4 C/T and 2 TMC4 T/T); in the three mutation groups we enrolled 6 patients with PNPLA3, TM6SF2, and MBOAT7 mutations (n. 3: I148I/M and 3 I148M/M; n. 4: 167E/K and 2: 167K/K; n. 2 TMC4 C/T and 4 TMC4 T/T).

**Figure 2:**
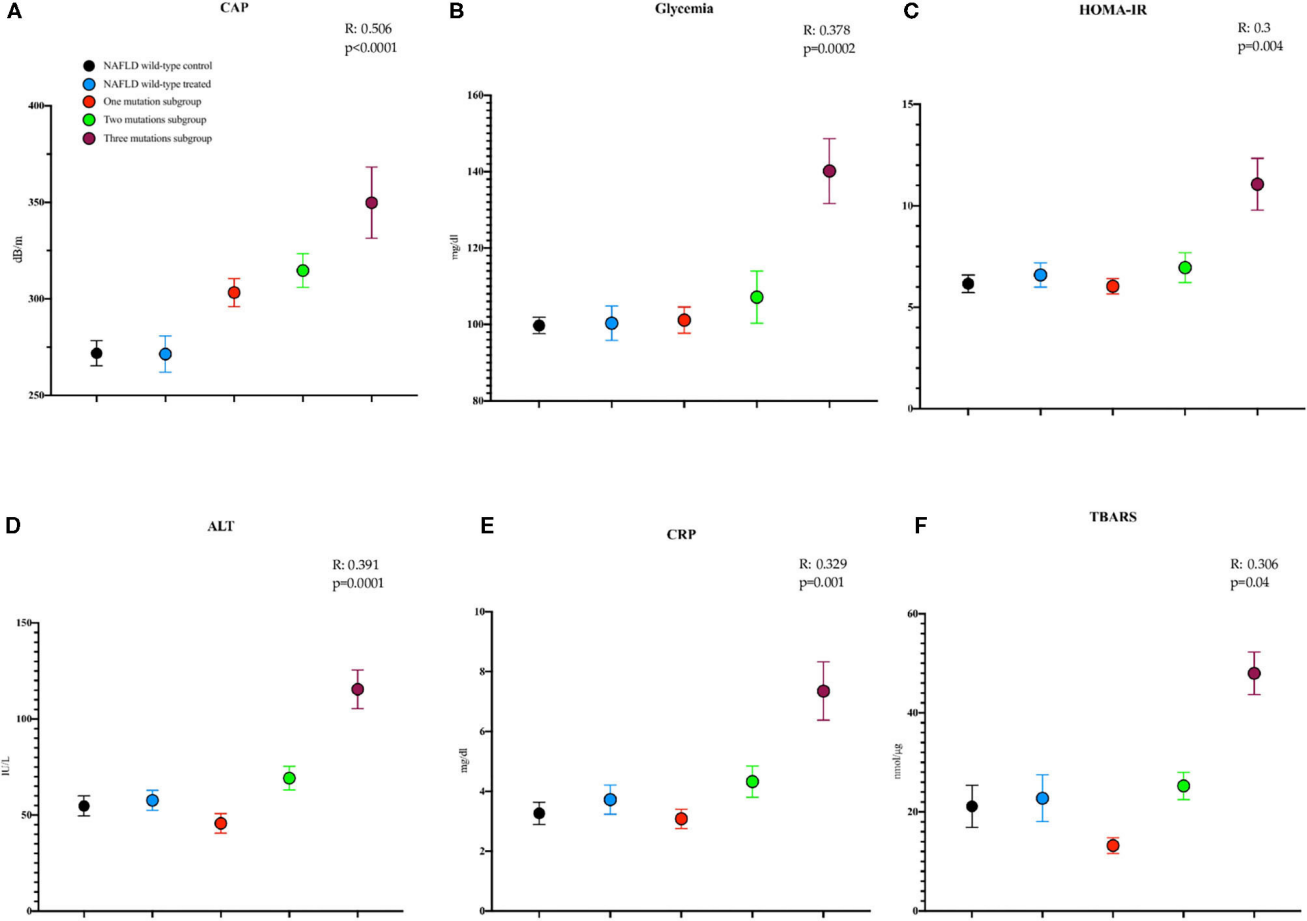
Mean with SEM values of some study parameters showing, at the linear regression model applied, a direct worsening relationship with the number of mutations at baseline. **(A)** Controlled attenuation parameter (CAP) (NAFLD wild type control: T0: 271.9 ± 6.5; NAFLD wild type treated: T0: 271.5 ± 9.4; one mutation subgroup: T0: 303.3 ± 7.3; two mutations subgroup: T0: 314.7 ± 8.7; three mutations subgroup: T0: 349.8 ± 18.5 dB/m). **(B)** Glycemia (NAFLD wild type control: T0: 99.7 ± 2.1; NAFLD wild type treated: T0: 100.3 ± 4.5; one mutation subgroup: T0: 101.2 ± 3.4; two mutations subgroup: T0: 107.2 ± 6.8; three mutations subgroup: T0: 140.2 ± 8.5, mg/dl). **(C)** The homeostatic model assessment for insulin resistance (HOMA-IR) (NAFLD wild type control: T0: 6.15 ± 0.4; NAFLD wild-type treated: T0: 6.59 ± 0.59; one mutation subgroup: T0: 6.03 ± 0.37; two mutations subgroup: T0: 6.95 ± 0.73; three mutations subgroup: T0: 11.06 ± 1.28). **(D)** Alanine aminotransferase (ALT) (NAFLD wild-type control: T0: 54 ± 5.2; NAFLD wild-type treated: T0: 57 ± 5.1; one mutation subgroup: T0: 45 ± 5.1; two mutations subgroup: T0: 69 ± 6.1; three mutations subgroup: T0: 115 ± 10 IU/L). **(E)** C reactive protein (CRP) (NAFLD wild type control: T0: 3.26 ± 0.37; NAFLD wild type treated: T0: 3.72 ± 0.48; one mutation subgroup: T0: 3.08 ± 0.32; two mutations subgroup: T0: 4.32 ± 0.59; three mutations subgroup: T0: 7.34 ± 0.97 mg/dl). **(F)** The thiobarbituric acid reactive substance (TBARS) (NAFLD wild-type control: T0: 21.12 ± 4.26; NAFLD wild-type treated: T0: 22.75 ± 4.73; one mutation subgroup: T0: 13.21 ± 1.61; two mutations subgroup: T0: 25.25 ± 2.76; three mutations subgroup: T0: 47.97 ± 4.29 nmol/μg).

Here we highlighted a direct linear correlation between the worsening of some of the baseline evaluated parameters and the number of concomitant mutations as shown by the linear regression applied: CAP (R: 0.506, *p* < 0.0001), glycemia (R: 0.378, *p* = 0.0002), HOMA-IR (R: 0.3, *p* = 0.004), ALT (R: 0.391, *p* = 0.0001), CRP (R: 0.329, *p* = 0.001), and TBARS (R: 0.306, *p* = 0.04) (Figure 2).

Moreover, we found a direct worsening correlation between the number of mutations and both the baseline and end of treatment values regarding most of the evaluated parameters: CAP (Pearson: p <0.0001, both T0 and T6), insulinemia (Pearson: *p* = 0.01 T6), FPG (Kendall Tau-b: *p* = 0.03 and 0.003, T0 and T6, respectively), HOMA-IR (Kendall Tau-b: *p* = 0.001 T6), ALT (Kendall Tau-b: *p* = 0.008 and 0.0002 T0 and T6, respectively), CRP (Kendall Tau-b: *p* = 0.001 and 0.0002 T0 and T6, respectively), and TBARS (Kendall Tau-b: *p* = 0.001 and 0.01 T0 and T6, respectively).

Assessing the specific responses among the groups with one, two, or three mutations we found only a statistically significant improvement of the TBARS assessment between baseline and the end of treatment in one mutation group (T0: 13.21 ± 5.81, T6: 9.94 ± 4.28, *p* = 0.002). None of the evaluated parameters showed significant improvement at the end of treatment in comparison to the baseline in both two and three mutation groups (Supplementary Tables 2–4).

## Discussion

Nowadays NAFLD represents the most important liver illness of the Western countries, not only for obvious epidemiological reasons but also for the growing scientific evidence of a fast natural history evolution and the burden of the optimum medical management that remains a difficult challenge of modern hepatology (26, 27). In this complex clinical scenario, the GWAS shed light on the role of several allelic variants in the NAFLD development and worsening (11).

It is also reasonable to hypothesize an effect produced by these allelic variants in the regulation of the response to NALFD therapy, due to their strong involvement in this pathological picture (28).

As shown in other clinical trials, the use of silybin in NAFLD patients seemed to be able to exert some beneficial effects due to the well-known anti-inflammatory, antioxidant, and antifibrotic therapeutic properties (19, 21, 29).

A wide range of molecular mechanisms by which silybin exerts its biologic activities was already shown. In this regard, Trappoliere et al. (30), in an established *in vitro* model of human hepatic fibrogenesis, demonstrated direct and indirect anti-fibrotic properties of silybin by reducing platelet derived growing factor (PDGF)-induced cell proliferation and migration. Salomone et al. investigated whether silybin may exert hepatoprotective effects by modulating nicotinamide adenine dinucleotide (NAD+) homeostasis and the SIRT1/AMP-activated protein kinase (AMPK) pathway (31). It was highlighted that silybin restores NAD+ levels and induces the SIRT1/AMPK pathway *in vitro* and *in vivo* (31).

In our previous study, the beneficial effect for NAFLD patients that could derive from the use of 6 months of oral administration of 303 mg of silybin-phospholipid complex, 10 μg of vitamin D, and 15 mg of vitamin E twice a day, on some metabolic, oxidative stress parameters as well as endothelial dysfunction ones was highlighted (19).

As noticed, in our current clinical setting, only the wild type treated group showed a statistically significant improvement of FPG, insulinemia, HOMA-IR, ALT, CRP, and TBARS; conversely, the NAFLD wild type control one showed, after 6 months, a worsening of TBARS in comparison to the baseline assessment. The logistic regression analysis confirmed that, for all the improved parameters, the patients' genotype plays a crucial role in the obtainment of useful therapeutic outcome, independently from other confounding variables: age, sex, comorbidities, medications, liver stiffness, and CAP.

It is important to notice that for this observation our results highlighted the relative independence to the specific genotype for the lack of therapeutic effect. In other words, the presence of at least one of the abovementioned mutations was demonstrated sufficiently able to determine the loss of useful therapeutic outcome.

In our knowledge, it is the first observation of an independent involvement of these genetic variants in regulating the response to a silybin-based therapeutic approach, even if their involvement in other therapeutic strategies was already proven (32, 33). It was highlighted, in 154 NAFLD adult Hong Kong residents, a PNPLA3 involvement in regulating the response to 30 min per day of physical exercise and an individually designed dietary regimen oriented to enhance the consumption of fruit, vegetables, moderate carbohydrates, low fat, low glycemic index, and low-caloric products, in accordance with the recommendations of the American Dietetic Association (34).

NAFLD patients, specifically G-allele carriers, demonstrated a greater reduction in intrahepatic triglyceride content (GG: 11.3 ± 8.8%) compared to those carrying the C-allele (CC: 3.7 ± 5.2%, CG: 6.5 ± 3.6%) after 12 months of treatment, together with an improvement of body weight, WHtR, total cholesterol, and low-density lipoproteins but without biochemical and liver stiffness changes (34).

This observation seems to suggest a possible way to counteract the harmful effect due to the PNPLA3 genetic predisposition to NAFLD, recommending a low caloric, well-balanced diet regimen and physical exercise, even if the effect of these therapeutic approaches needs to be assessed in depth with other large population clinical trials, also considering the possibility of a different outcome in the case of concomitant multiple gene mutations. On the other hand, the genetic influence on the pharmacological therapy outcome seems to be different. “The WELCOME trial” assessed the response to omega-3 high dosage therapeutic intervention on liver fat content and fibrosis in NAFLD patients (35). Fifty-one patients received Omacor® 4 g/d (4 × 1,000 mg capsules of 460 mg eicosapentaenoic acid and 380 mg docosahexaenoic acid) for 15–18 months. Fifty-two NAFLD patients were treated with a placebo of isocaloric olive oil (4 g/d) containing ~67% oleic acid, 15% linoleic acid, 15% palmitic acid, 2% stearic acid, and 1% alpha linolenic acid (35). At the end of treatment, those patients carrying the 148I/I and 148I/M genotype showed a decrease of liver fat percentage (148I/I: −7.05%, 148I/M: −7.30%) whereas the 148M/M group showed a moderate increase (2.75%) (35). Similar findings were obtained from an obese related pediatric Italian NAFLD population, demonstrating the involvement of the PNPLA3 in regulating the response to this pharmacologic intervention (36).

Currently the scientific literature lacks data regarding the involvement of TM6SF2 and MBOAT7 genes on NAFLD therapy outcomes.

Here we highlighted that independently of having one, two, or three mutations, the patients did not show the same improvements recognized in the wild type group.

The main involvement of these genes in the hepatic fat accumulation could give the possible explanation for the associated insulin resistance that in turn represents one of the causes but also the effect of the hepatic steatosis and worsening fibrosis in NAFLD context as well as the linked systemic inflammation that typically affect this category of subjects determining higher TBARS and CRP levels (19, 37, 38). The lack of the therapeutic response in the NAFLD treated mutated group could be related to the impact that these genetic variants could have in inducing precisely hepatic fat accumulation, that in turn is responsible itself for the worsening of the clinical picture and, moreover, could act by reducing the potential useful effect induced by the described therapeutic regimen, whose efficiency in the light of the results shown could need to be reassessed in large randomized controlled trials considering the genetic influence on interpreting the results. The lack of a therapeutic effect on liver stiffness and CAP as well, in the light of the results shown, is not surprising due to the fact that, as properly demonstrated in a previously published randomized controlled trial showing the histologic improvement of liver inflammation and fibrosis after 12 months of therapy, a longer administration schedule could be necessary for the obtainment of more important results in wild type patients (21).

Our study has the limitation to not have been assessed the specific response rate dividing the patients in accordance with the homozygosis or heterozygosis genotype as well as the specific gene effect. However, it was not identified as the endpoint of the current study because there is no other scientific knowledge assessing the comparison between wild type patients and mutated ones on the response to nutraceutical therapy.

The next future prospective could be to increase the sample size of the subgroups in accordance with the specific mutated genes and the homozygosis or heterozygosis genotype, as well as proceed to design a large clinical trial exploring the histologic improvement that could derive from the therapeutic regimen randomizing the population in accordance with the specific PNPLA3, TM6SF2, and MBOAT7 genotype.

## Data Availability Statement

The original contributions presented in the study are included in the article/Supplementary Material, further inquiries can be directed to the corresponding author/s.

## Ethics Statement

The studies involving human participants were reviewed and approved by the Ethical Committee of the University of Campania L. Vanvitelli in Naples. The patients/participants provided their written informed consent to participate in this study.

## Author Contributions

MD: guarantor of the article, conceptualization, methodology, formal analysis, investigation, and writing—original draft. MM and FM: investigation. MR: formal analysis. CT: methodology and resources. MP: visualization and supervision. CL: resources, data curation, and visualization. AF: conceptualization, data curation, supervision, and project administration. All authors approved the final version of the manuscript.

## Conflict of Interest

The authors declare that the research was conducted in the absence of any commercial or financial relationships that could be construed as a potential conflict of interest.

## Publisher's Note

All claims expressed in this article are solely those of the authors and do not necessarily represent those of their affiliated organizations, or those of the publisher, the editors and the reviewers. Any product that may be evaluated in this article, or claim that may be made by its manufacturer, is not guaranteed or endorsed by the publisher.
