# Supplementary material for: PNPLA3, TM6SF2, and MBOAT7 Influence on Nutraceutical Therapy Response for Non-alcoholic Fatty Liver Disease: A Randomized Controlled Trial
Source: Front Med (Lausanne). 2021 Oct 8;8:734847. doi: 10.3389/fmed.2021.734847 (PMC8531439; doi:10.3389/fmed.2021.734847)
Supplement: Supplementary file 1 [file Table_1.DOCX]

**Table S1: Baseline and end of treatment daily intake assessment among the three study groups**

| **Variables (M±SD)** | **NAFLD wild-type control Group**  **(*n.* 30)** | | | **NAFLD wild type treated group**  **(*n.* 30)** | | | **NAFLD mutated treated group**  **(*n.* 32)** | | |  |
| --- | --- | --- | --- | --- | --- | --- | --- | --- | --- | --- |
|  | **Baseline** | **End of treatment** | **p** | **Baseline** | **End of treatment** | **p** | **Baseline** | **End of treatment** | **p** | **p value of the baseline comparison among the groups** |
| Daily intake (K-cal) | 1473±566.6 | 1471±507.8 | 0.726 | 1479±601.3 | 1475±436.1 | 0.539 | 1452±586.5 | 1474±515.1 | 0.214 | Mutated vs WT treated: >0.999  Mutated vs WT control: >0.999  WT treated vs WT control: >0.999 |
| Proteins (K-cal) | 226±66.8 | 221.7±145.7 | 0.587 | 211.3±78.2 | 204.7±98.2 | 0.15 | 210.2±94.9 | 213.3±132.2 | 0.407 | Mutated vs WT treated: >0.999  Mutated vs WT control: 0.346  WT treated vs WT control: 0.522 |
| Lipids (K-cal) | 502.6±246.2 | 501.4±191.6 | 0.726 | 497.1±215.5 | 499.1±181.1 | 0.741 | 485.8±213.3 | 488.2±170.6 | 0.988 | Mutated vs WT treated: >0.999  Mutated vs WT control: >0.999  WT treated vs WT control: >0.999 |
| Carbohydrates (K-cal) | 743.9±299.5 | 748.2±230.8 | 0.983 | 770.1±340.7 | 771.6±231.1 | 0.519 | 755.6±323.7 | 772.8±272.5 | 0.077 | Mutated vs WT treated: >0.999  Mutated vs WT control: >0.999  WT treated vs WT control: >0.999 |

For the comparison of the baseline and end of treatment values in each group, Wilcoxon signed ranks test and t-test for dependent groups were performed according to non-normal and normal distribution respectively.

The Kruskal-Wallis test or ANOVA test with post-hoc Bonferroni analysis, in case of non-normal or normal distribution respectively, were performed to compare the continuous variables among three groups.
