# Supplementary material for: PNPLA3, TM6SF2, and MBOAT7 Influence on Nutraceutical Therapy Response for Non-alcoholic Fatty Liver Disease: A Randomized Controlled Trial
Source: Front Med (Lausanne). 2021 Oct 8;8:734847. doi: 10.3389/fmed.2021.734847 (PMC8531439; doi:10.3389/fmed.2021.734847)
Supplement: Supplementary file 2 [file Table_2.docx]

| **Variables (M±SD)** | **Baseline** | **End of treatment** | **p** |
| --- | --- | --- | --- |
| BMI (kg/m2) | 28.6±2.2 | 29.4±2.6 | 0.057 |
| WHtR | 1.05±0.19 | 1.05±0.19 | 0.709 |
| CAP (dB/m) | 303.3±26.3 | 283.3±36.7 | 0.065 |
| Stiffness (kPa) | 4.3±1 | 3.9±1.6 | 0.25 |
| FPG (mg/dl) | 101.2±12.4 | 101.4±15 | 0.891 |
| Insulinemia (μU/ml) | 22.4±5.26 | 22.01±4.24 | 0.305 |
| HOMA-IR | 6.03±1.34 | 5.51±1.49 | 0.424 |
| AST (IU/L) | 39±34 | 30±16 | 0.986 |
| ALT (IU/L) | 45±18 | 39±14 | 0.338 |
| GGT (IU/L) | 71±83 | 74±72 | 0.386 |
| CRP (mg/dl) | 3.08±1.16 | 2.93±1.16 | 0.786 |
| TBARS (nmol/μg) | 13.21±5.81 | 9.94±4.28 | 0.002 |

**Table S2: Baseline and end of treatment evaluation of stiffness, anthropometric, metabolic and biochemical parameters of patients with one mutation.**

BMI: body mass index; WHtR: waist-to-height ratio; CAP: controlled attenuation parameter; FPG: fasting plasma glucose; HOMA-IR: homeostatic model assessment for insulin resistance; GGT: gamma-glutamyl transferase; AST: aspartate aminotransferase; ALT: alanine aminotransferase; CRP: C reactive protein; TBARS: thiobarbituric acid reactive substances.

For the comparison of the therapeutic outcome in each group for the continuous variables, wilcoxon signed ranks test and t-test for dependent groups were performed according to non-normal and normal distribution respectively.
