# Supplementary material for: PNPLA3, TM6SF2, and MBOAT7 Influence on Nutraceutical Therapy Response for Non-alcoholic Fatty Liver Disease: A Randomized Controlled Trial
Source: Front Med (Lausanne). 2021 Oct 8;8:734847. doi: 10.3389/fmed.2021.734847 (PMC8531439; doi:10.3389/fmed.2021.734847)
Supplement: Supplementary file 3 [file Table_3.DOCX]

| **Variables (M±SD)** | **Baseline** | **End of treatment** | **p** |
| --- | --- | --- | --- |
| BMI (kg/m2) | 31.3±4.5 | 31.5±3.7 | 0.892 |
| WHtR | 1.08±0.25 | 1.08±0.26 | 0.65 |
| CAP (dB/m) | 314.7±31.5 | 312.8±45.6 | 0.791 |
| Stiffness (kPa) | 5.1±1.7 | 5.4±1.4 | 0.28 |
| FPG (mg/dl) | 107.2±24.6 | 103.2±25 | 0.519 |
| Insulinemia (μU/ml) | 26±6.2 | 25.1±5.8 | 0.717 |
| HOMA-IR | 6.95±2.65 | 6.6±2.9 | 0.677 |
| AST (IU/L) | 39±42 | 40±46 | 0.931 |
| ALT (IU/L) | 69±22 | 66±34 | 0.892 |
| GGT (IU/L) | 45±36 | 53±65 | 0.691 |
| CRP (mg/dl) | 4.32±1.88 | 4.26±2.14 | 0.647 |
| TBARS (nmol/μg) | 25.25±9.95 | 23.52±14.78 | 0.959 |

**Table S3: Baseline and end of treatment evaluation of stiffness, anthropometric, metabolic and biochemical parameters of patients with two mutations.**

BMI: body mass index; WHtR: waist-to-height ratio; CAP: controlled attenuation parameter; FPG: fasting plasma glucose; HOMA-IR: homeostatic model assessment for insulin resistance; AST: aspartate aminotransferase; ALT: alanine aminotransferase; GGT: gamma-glutamyl transferase; CRP: C reactive protein; TBARS: thiobarbituric acid reactive substances.

For the comparison of the therapeutic outcome in each group for the continuous variables, wilcoxon signed ranks test and t-test for dependent groups were performed according to non-normal and normal distribution respectively.
