# Supplementary material for: PNPLA3, TM6SF2, and MBOAT7 Influence on Nutraceutical Therapy Response for Non-alcoholic Fatty Liver Disease: A Randomized Controlled Trial
Source: Front Med (Lausanne). 2021 Oct 8;8:734847. doi: 10.3389/fmed.2021.734847 (PMC8531439; doi:10.3389/fmed.2021.734847)
Supplement: Supplementary file 4 [file Table_4.docx]

| **Variables (M±SD)** | **Baseline** | **End of treatment** | **p** |
| --- | --- | --- | --- |
| BMI (kg/m2) | 32.7±5.2 | 33.7±4.1 | 0.218 |
| WHtR | 1.15±0.38 | 1.14±0.38 | 0.312 |
| CAP (dB/m) | 349.8±45.2 | 337.1±29 | 0.562 |
| Stiffness (kPa) | 5.1±1.6 | 5.1±1.7 | 0.562 |
| FPG (mg/dl) | 140.2±20.8 | 148.2±13.2 | 0.156 |
| Insulinemia (μU/ml) | 31.6±6.1 | 32.4±8.5 | 0.843 |
| HOMA-IR | 11.06±3.14 | 11.97±3.71 | 0.218 |
| AST (IU/L) | 32±9 | 26±9 | 0.312 |
| ALT (IU/L) | 115±24 | 134±33 | 0.062 |
| GGT (IU/L) | 84±122 | 66±73 | 0.687 |
| CRP (mg/dl) | 7.34±2.38 | 7.89±1.65 | 0.437 |
| TBARS (nmol/μg) | 47.97±10.51 | 51.49±10.57 | 0.093 |

**Table S4: Baseline and end of treatment evaluation of stiffness, anthropometric, metabolic and biochemical parameters of patients with three mutations.**

BMI: body mass index; WHtR: waist-to-height ratio; CAP: controlled attenuation parameter; FPG: fasting plasma glucose; HOMA-IR: homeostatic model assessment for insulin resistance; AST: aspartate aminotransferase; ALT: alanine aminotransferase; GGT: gamma-glutamyl transferase; CRP: C reactive protein; TBARS: thiobarbituric acid reactive substances.

For the comparison of the therapeutic outcome in each group for the continuous variables, wilcoxon signed ranks test and t-test for dependent groups were performed according to non-normal and normal distribution respectively.
